# Systematic Review of Tools and Methods to Measure Appetite in Undernourished Children in the Context of Low- and Middle-Income Countries

**DOI:** 10.1093/advances/nmy042

**Published:** 2018-11-21

**Authors:** Scott B Ickes, Muttaquina Hossain, Gaelen Ritter, Monica Lazarus, Katie Reynolds, Baitun Nahar, Tahmeed Ahmed, Judd Walson, Donna M Denno

**Affiliations:** 1 Departments of Health Services; 2 Pediatrics; 3 Global Health; 4 Medicine; 5 Epidemiology; 6 Program in Nutritional Sciences, University of Washington, Seattle, WA; 7 Nutrition and Clinical Services Division, International Centre for Diarrheal Diseases Research, Bangladesh (icddr,b); 8 Childhood Acute Illness and Nutrition Network, Nairobi, Kenya; 9 Wheaton College Department of Applied Health Sciences, Wheaton, IL

**Keywords:** appetite, caregiver perceptions, dietary assessment, environmental enteric dysfunction, stunting, undernutrition

## Abstract

Child undernutrition has multifactorial causes, ranging from food insecurity to etiologies refractory to conventional nutritional approaches, such as infections, environmental enteric dysfunction, and other conditions that lead to systemic inflammation. Poor appetite may be an important symptom of these causes and may be a useful marker of an undernourished child's ability to recover. We conducted a systematic review to characterize the methods and tools to measure appetite among children <5 y old in low- and middle-income countries. A systematic search of 8 databases identified 23 eligible studies published since 1995. Thirteen described methods based on direct feeding observation or quantification of nutrient intake from caregiver report, 16 described tools that assessed caregiver perceptions of appetite, and 6 reported assessments in both categories. Four studies that gauged caregiver perceptions assessed multiple appetite domains, whereas 12 assessed 1 domain—often with a single question. Only 6 studies reported validation processes, the most common of which compared an observed test meal with daily energy intake. No studies reported the use of a method or tool that was validated in multiple cultural or linguistic contexts. Although dietary intake measures and observed feeding tests have shown validity in some contexts, they are resource intensive. Subjective caregiver questionnaires may offer a more efficient appetite evaluation method, but they have been evaluated less consistently. A rigorously developed and validated tool to rapidly assess child appetite is needed and could be best addressed by a questionnaire that leverages the multiple domains of appetite. The application of interventions that target causes of undernutrition that are not amenable to food-based interventions in clinical or research contexts could be facilitated by an efficient appetite screening tool to identify appetite-related causes of undernutrition and to monitor children's response to such interventions.

## Introduction

Every year nearly 5.6 million children in low- and middle-income countries (LMICs) die, most from preventable causes (1). Undernutrition is an underlying cause for ∼45% of these deaths and is due to a variety of factors that include food insecurity, inadequate care, and lack of responsive feeding (2). However, <40% of the variance in linear growth retardation in children can be attributed to diet (3). Furthermore, complementary feeding interventions to improve dietary adequacy address only approximately one-third of the linear growth deficit among children <2 y old in LMICs (4). Studies in multiple settings have reported that undernourished children often do not consume offered food as would be expected, suggesting that food availability is not solely responsible for poor dietary intake (5, 6). One hypothesis for poor intake among these children is anorexia or poor appetite, and thus appetite has been measured as an outcome in various nutrition and health interventional studies.

Beyond issues with caregiving and food security, malnutrition is caused by a number of underlying conditions that often result from acute, recurrent, and chronic illness, including immune activation, impaired nutrient absorption, dysregulated metabolism, disruption in hormonal response, and systemic and local inflammation (2, 7, 8). For example, environmental enteric dysfunction (EED) is thought to be a potentially important cause of malnutrition, especially stunted linear growth, defined as length-for-age <−2 *z* scores. EED, a condition prevalent in resource-poor settings with fecally contaminated environments, is characterized by subclinical chronic gut inflammation leading to blunting of the villous lining of the small intestine, reduced nutrient absorption, intestinal permeability and translocation of microbes or microbial products, and resultant systemic inflammation (9). Impaired metabolism and systemic inflammation as a result of EED or other causes of systemic inflammation and activation are associated with poor appetite and reduced energy intake, which can perpetuate a cycle of undernutrition, including stunted growth and poor health (10–12). These pathophysiologic processes can contribute to poor ponderal growth and decreased linear growth due to effects on growth-plate ossification (13).

Children experiencing growth faltering due to nonnutritional causes, such as abnormal metabolism or systemic inflammation, may experience minimal or no recovery in response to nutritional interventions such as supplemental or therapeutic feeding. Poor appetite accompanying these nonnutritional causes of stunted growth is not assessed as part of routine child health care in LMICs (14, 15). In such contexts, a simple, noninvasive method to screen for poor appetite as a surrogate for chronic inflammation may be helpful to identify children for whom food-based approaches alone may fail to improve nutritional status and whose underlying medical conditions may require targeted interventions. Such assessments could be useful in stratifying undernourished children, including those with poor linear growth, for interventions targeting specific underlying conditions and mechanisms and could also be used to follow response to treatment.

As a complex emotional, biological, and social phenomenon, child appetite has been variably defined and measured. Some definitions describe appetite as a process that guides food selection (16), whereas others connect it to the physiologic experience of hunger that is directed at food choices with expectations of reward (17, 18). In light of the potential utility of applications of child appetite measurement, we conducted a systematic review of peer-reviewed literature to *1*) describe currently used methods for child appetite assessment in LMICs and *2*) to analyze these methods to identify opportunities for improving appetite measurement in this population.

## Methods

This review sought to assess what tools and methods have been used to measure appetite among children <5 y old in LMICs. Although we initially sought to identify and analyze only validated tools, it became apparent early in the review that too few validated tools would be retrieved; therefore, the search was expanded to include both validated and nonvalidated assessments.

We conducted a systematic search of PubMed (http://www.pubmed.com), CAB Direct (https://www.cabdirect.org), EMBASE (https://www.embase.com), and the 5 WHO regional databases (http://www.globalhealthlibrary.net/php/index.php) for articles published between 1995 and December 2016. We also used a snowball technique to identify additional relevant references from retrieved sources.

In collaboration with a health sciences librarian, we developed unique Boolean expressions (**Supplemental Table 1**) for each database. Two of 4 investigators (MH, ML, GR, and KR) reviewed each search result against the inclusion and exclusion criteria as listed in **Table 1**. Briefly, these criteria required that articles describe a measurement of appetite with the use of a tool, method, or single question among children in an LMIC. At least a portion of the study's assessment of appetite must have included children aged 0–4 y. Exclusion criteria included investigations conducted outside of an LMIC setting or in the context of overnutrition, developmental disability, or medical conditions that interfere with oral-motor feeding. Articles published in English, Spanish, French, or Portuguese were included. Studies whose assessment of appetite was not part of their specific aim were retained as long as the appetite assessment was sufficiently described. If an assessment of the suitability of the article was indeterminate on the basis of the abstract and title, the full-text article was sought.

**TABLE 1:** Article inclusion and exclusion criteria for database searches

Inclusion criteria
1. Measures of appetite using a tool or method in a low- or middle-income country^1^
2. Included measures of appetite among children 0–4 y of age
Exclusion criteria
1. Does not measure appetite or does not specify the measurement process
2. Measures appetite, but in the context of overnutrition or emotional eating
3. Measures appetite, but in the context of developmental disability or medical condition that interferes with oral-motor feeding
4. Measure of appetite was a biomarker tested on blood or other bodily specimen
5. Measurement was not conducted in a low- or middle-income country^1^
6. Measurement was not in the 0- to 5-y age population
7. Article was not in English, Spanish, French, or Portuguese
8. Study was conducted before 1995

^1^Per the World Bank designation, the year the study was published. Data are available at: https://datahelpdesk.worldbank.org/knowledgebase/articles/906519-world-bank-country-and-lending-groups.

Due to a lack of a uniformly accepted definition of child appetite in the literature, we accepted the author definitions of appetite and accepted the term “anorexia” to be equivalent to poor appetite. For the purposes of this review, an appetite domain was defined as a sphere of knowledge or behavior pertaining to child appetite (e.g., a child's interest in food, the amount of food a child consumes compared with his or her usual intake); a scale is the numeric measurement of a domain; and a subscale is the numeric measurement of a domain where multiple domains are assessed. We also defined “validated” as a second, objective measurement of appetite that was used to compare the values of the index appetite measurement.

We tracked the total number of publications retrieved from each database combined, excluding duplicates. Discrepancies between research assistants’ inclusion and exclusion determinations were uncommon (<5% of the total included articles) and diminished as the process progressed. When needed, 2 investigators (SBI and DMD) arbitrated the differences in determinations during meetings with the research team.

**FIGURE 1:**
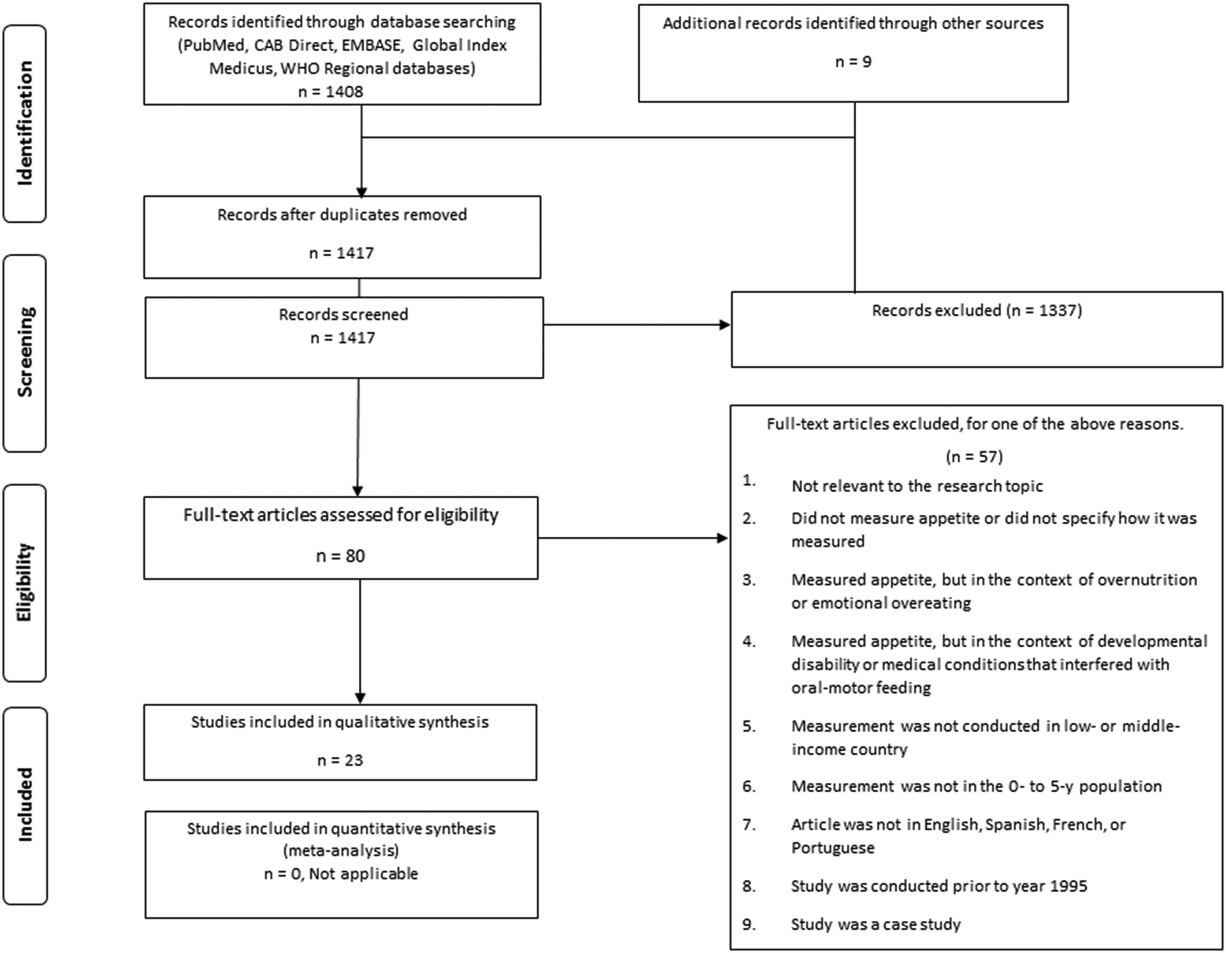
PRISMA flowchart of article inclusion and exclusion criteria based on study protocol. PRISMA, Preferred Reporting Items for Systematic Reviews and Meta-Analyses.

We conducted the review methods and reporting procedures in accordance with the PRISMA (Preferred Reporting Items for Systematic Reviews and Meta-Analyses) statement (19) and registered the review with PROSPERO (no. 49806). As summarized in **Figure 1****,** the PRISMA flow diagram, the search from the 8 databases yielded 1417 unique publications. Of these, 80 articles were identified as potentially meeting inclusion criteria on the basis of the article title or abstract. On the basis of assessment of the full text of these 80 articles, an additional 57 were excluded.

## Results

Twenty-three studies were included in the final review (Figure 1), of which 13 were classified as using tools or methods that measured appetite by quantifying dietary intake, either through direct observation or caregiver recall of food consumption (**Table 2**), and 16 were categorized as using tools or methods that assessed child appetite through caregiver perceptions (**Table 3**). Six studies were included in both categories (20–23, 25, 27).

**TABLE 2:** Evidence table: tools and measures that assessed child appetite through direct observation of dietary intake or quantification of intake based on caregiver record^1^

Study (reference)	Study design and objective	Setting, sample size, and population	Method of appetite assessment	Methods of validation
Brown et al. (20)	Cohort study to assess the validity of maternal reports of poor infant appetite.	Peru. Measured caregiver report of anorexia (*n* = 153) and energy consumption (*n* = 131) of singleton newborns weighing ≥2500 g at birth living in an economically disadvantaged periurban community outside of Lima. Children followed for 1 y, for 1621 d of child observations. No exclusion criteria or baseline morbidity data were reported.	In-home dietary intake was assessed on 1 or 2 d/mo by means of direct observation and weighing all foods and breast milk consumed during the daytime hours. Nighttime intakes were obtained by recall history and by extrapolation of the amount of breast milk consumed during 12 daytime hours. Energy and nutrient intakes were converted using food-composition tables and direct measurement of proximate components of breast-milk samples. Based on caregiver report of anorexia (described in Table 3), energy intake on days with and without anorexia were compared.	Maternal perception of appetite assessment was validated against energy intake calculations. See Table 3.
			Energy intake results were presented in relation to maternal reports of child appetite and are presented in Table 3.	
			No definition of appetite level based on energy intake was reported.	
Ciliberto et al. (21)	Case series to determine the safety and effectiveness of outpatient management of edematous malnutrition.	Malawi. A total of 219 children aged 12–60 mo with mild edematous malnutrition and good appetite were enrolled. Mean ± SD HAZ was −2.9 ± 1.4; WHZ was −1.7 ± 0.9.Children were drawn from 2 large, previously reported, community-based malnutrition studies. Children aged 12–60 mos who presented to 1 of 7 nutritional rehabilitation units or 1 of 8 villages in a community-based prevention trial were screened.	Assessment was conducted at a research site close to participant homes and consisted of observation of child consuming a test dose of 30 g RUTF. Good appetite was determined if the child consumed 100% of the RUTF within 20 min of observation.Nine children were excluded from enrollment in this study because they “did not consume the test dose of RUTF.”Caregiver report of child's usual food consumption was also assessed (see Table 3).	NA
Cohen et al. (22)	RCT to assess food acceptance based on timing of solid food introduction. Women were visited weekly during the first 4 mo postpartum. At 16 wk, they were randomly assigned to exclusive breastfeeding until 26 wk, solid foods with ad libitum breastfeeding, or solid foods plus breastfeeding as often as before.	Honduras. A total of 141 primiparous mothers of newborns willing to exclusively breastfeed for 26 wk. Infants were healthy, full-term, and with a birth weight ≥2000 g.Mothers were aged ≥16 y, low-income, not employed outside the home, and living in conditions of poor environmental sanitation.	In-home food intake was measured by study staff among a subsample at 9-mo (*n* = 60) and 12-mo (*n* = 123) postintervention visits. Caregivers were notified of the week but not the day of the home visit to avoid bias. Mothers were provided with food grinders to make home-prepared baby foods. The amount of food offered and consumed at the midday meal was measured. Investigators calculated grams of food consumed, and percentage of food consumed from the amount offered, not including beverages.	NA
			No definition of appetite level was reported.Investigators found that the mean unconsumed food at the midday meal was >40% at 9 mo and 25% at 12 mo and did not significantly differ between intervention arms.Caregiver report of child's food acceptance was also assessed (see Table 3).	
Dossa et al. (23)	RCT to assess whether a combination of MVMM supplement and additional iron treatment improved the appetite, morbidity status, hemoglobin, and growth of children over 6 wk of supplementation.	Benin. A total of 154 children aged 18–30 mo who were stunted (HAZ <−2) and anemic (hemoglobin <110 g/L) were randomly assigned to 1 of 4 arms (4 children lost to follow-up): MVMM/Fe (*n* = 39); MVMM/placebo (*n* = 37); placebo/Fe (*n* = 35); placebo/placebo (*n* = 39). Overall baseline mean HAZ was −2.6 ± 0.7 and did not differ between groups.Exclusion criteria included: HAZ <−5; not liking rice or do not eat rice per maternal report.	Two relevant assessments:1. In-home dietary intake was measured as per Cameron and van Staveren (24) on 3 consecutive days by study staff among a subset of 38 children. Staff measured the weight of all individual foods consumed over 12–14 h. Few children reportedly consumed food overnight, in which case food consumption was per caretaker recall. Breakfast and daily energy intakes were calculated using nutrient-composition software.2. An observed feeding of a breakfast test meal was conducted on days not overlapping with the above-described assessments. Caregivers were instructed to fast children from the last meal of the previous day until the morning test feeding, which was performed under research staff supervision (setting not specified) with individual mother-child pairs. Children were first offered 250 g of a liquid test food of fermented maize porridge (*aklui*) or solid test food of rice cooked with tomato sauce (*riz-au-gras*); mothers were instructed to feed children ad libitum, but without inducement. Another 250 g was offered if child asked for more. After the child stopped eating, a 5-min break was given, followed by instructions to continue feeding. Three feeding episodes were offered.The amount of test food consumed, duration of feeding, and food intake per minute were calculated for the first eating episode and for the sum of the 3 episodes.	Observed appetite test was validated against a 3-d dietary assessment intake. Calculated correlation between *1*) energy intake from appetite test and *2*) 3-d food intake. Energy intake of test food (appetite test) was significantly correlated with 3-d breakfast energy intake before (*r* = 0.49, *P* = 0.002) and after (*r* = 0.42, *P* = 0.008) supplementation. Correlations between the test food energy intake (appetite test) and 3-d, 24-h energy intake were weaker and not significant compared with the breakfast-only intake before (*r* = 0.22, *P* = 0.18) and after (*r* = 0.26, *P* = 0.12) supplementation.
			No definition of appetite level was reported.	
			The total energy intake (mean ± SD) from the test food increased significantly in all treatment (MVMM) groups after supplementation (MVMM/Fe = 988 ± 519 kJ before treatment vs. 1275 ± 691 kJ after treatment; MVMM/placebo = 1037 ± 616 before treatment vs. 1264 ± 593 after treatment; *P* < 0.005). There was no significant difference between groups in changes in appetite (as measured by the appetite test) over the 6-wk supplementation period. The eating rate (measured in kJ/min) was higher (*P* < 0.05) in all groups except for the Fe/placebo group. Eating duration during the appetite test was unchanged from baseline in all groups, except for MVMM/placebo, which increased.	
			Caregiver report of child's appetite was also assessed (see Table 3).	
Dossa et al. (25)	Cross-sectional study to determine if a controlled feeding assessment of a test food can be used as a reproducible and valid appetite tool for young children in field studies.	Benin. Total sample size of 109 children aged 18–30 mo. Four related studies were performed: study 1 (*n* = 8) was to test the methodology; studies 2 and 3 (*n* = 38, *n* = 38, respectively) elaborated on the methodology; study 4 (*n* = 25) included aspects of water intake in the assessment. Mean ± SE HAZ reported only in studies 1 and 4 and was 0.86 ± −0.66 and −1.73 ± 0.98, respectively. Mean WHZ was −0.62 ± 1.04 and −1.14 ± 0.77 in studies 1 and 4, respectively.No health-related inclusion or exclusion criteria were reported.	Two relevant assessments:1. In-home dietary intake: Weighed food records were obtained using the same method as described above for Dossa et al. (23).2. An observed feeding of a breakfast test meal as per Dossa et al. (23), except the offered test doses were 300 g at the first offering and 200 g if children requested more food. There were 2 feeding episodes in study 1, and 3 episodes in studies 2–4. The amount of test food consumed, feeding duration, and eating rate were calculated. The study setting was not specified.No definition of appetite level was reported.With *riz-au-gras* as the test food, there were positive correlation coefficients with the daily and breakfast energy intake, but the correlations were nonsignificant. With *aklui* as the test food, the coefficients were positive and significant (e.g., *r* = 0.41, *P* < 0.05 for study 2). Eating rate during the test meal was also positively correlated with total energy consumption based on the in-home dietary intake.	Observed test meal was validated against a 3-d dietary assessment intake in studies 1 and 3. Correlation coefficients were calculated as above. Energy intake from the observed test meal (based on the first eating episode and the total consumption for all episodes) was correlated with the energy intake of breakfast at home (*r* range = 0.40–0.52, *P* < 0.01, in study 3 only) and energy intake over 24 h at home (*r* range = 0.41–0.76, *P* < 0.05, in study 1; *P* < 0.01 in study 3). Also assessed correlations between eating rate of the test meal and energy intake of breakfast at home (−0.13 to 0.43, *P* < 0.01 in Study 3 only) and energy intake over 24 h at home (*r* range = 0.51–0.82, *P* < 0.05, in study 1 and *P* < 0.01 in study 3).
Dossa et al. (26)	RCT to assess effect of daily micronutrient supplementation on appetite and growth among stunted children. Anthropometric, appetite, and morbidity status were assessed weekly through 6 wk postintervention.	Benin. A total of 101 stunted (HAZ <−2) children aged 17–32 mo (mean ± SD HAZ = −2.8 ± 0.7) randomly assigned to MVMM treatment (*n* = 48) or placebo (*n* = 53). All participants needed to accept the fermented maize porridge used for control feeding.No health-related inclusion or exclusion criteria were reported.	Research site observed feeding of a breakfast test meal per Dossa (23, 25), except only *alkui* was used as the test food. The amount of test food consumed, feeding duration, and eating rate were calculated.The MVMM supplementation failed to improve appetite (assessed by energy consumption and eating duration and rate) and growth. Changes in grams of food consumed during the test meal were 27 ± 124 in treatment and 57 ± 129 in placebo. Changes in eating rate (g/min) were 6 ± 6 in treatment and 7 ± 9 in placebo.No definition of appetite level was reported.Caregiver report of child's appetite was also assessed (see Table 3).	No validation measures were described in this article, but the observed test meal used was nearly identical to Dossa et al. (23, 24). Validations in those studies are described above.
Khademian et al. (27)	RCT to assess effect of daily zinc administered for 12 wk on appetite and its subscales.	Iran. A total of 96 children aged 2–6 y with a chief complaint of anorexia were randomly assigned to zinc (*n* = 48) or placebo (*n* = 48); 78 children completed the trial (77 in zinc, 38 in placebo). For inclusion, anorexia could not be from a major organic cause (e.g., anemia, urinary tract infection, parasitic infection, hemoglobin <11 g/dL.). The nutritional status of children was not reported.	Maternal record of 3 d of children's food intake on weekdays. Total mean calorie, carbohydrate, protein, fat, and zinc intakes were estimated pre- and postsupplementation.Over 12 wk, mean ± SD energy intake decreased in the placebo group from 960.5 ± 22.2 kcal to 930 ± 22.4 kcal, and increased in the zinc group from 930 ± 136 to 1022 ± 160 kcal (*P* = 0.00).No definition of appetite level was reported.Caregiver report of child appetite was also assessed (see Table 3).	NA for the dietary intake portion of the study. Author-reported validation of CEBQ is described in Table 3.
Mda et al. (28)	RCT to assess the effect of multi-micronutrient supplementation on the appetite of children and their concentrations of appetite-regulating hormones.	South Africa. A total of 99 HIV-infected children aged 6–24 mo who had been previously admitted to hospital with pneumonia or diarrhea. Children were excluded if they were receiving ART, had received a micronutrient supplementation in the previous 2 mo, or had been diagnosed with a chronic illness unrelated to HIV infection. Mean ± SD HAZ (all participants) at enrollment was −1.27 ± 1.61; mean ± SD WHZ was −1.46 ± 1.24.	Observed feeding of a breakfast test meal based on Dossa et al. (25) was performed in a clinical setting at baseline and 3 and 6 mo. The test food used was a honey and wheat infant cereal (Nestle Nestum no. 2; 75 g dry porridge mixed with 480 mL milk).Amount of test food consumed, feeding duration, and eating rate were calculated and compared between intervention and placebo groups at baseline and 3 and 6 mo.The change in amount of test food eaten over the 6-mo follow up was significantly higher in the supplement group (101 ± 112 g compared with 57 ± 130 g in placebo group; *P* < 0.05). The change in amount of test food eaten per kilogram of body weight at the 6-mo follow-up was significantly higher in the supplement group (5.3 ± 14.9 g/kg compared with 4.7 ± 14.7 g/kg in placebo group; *P* < 0.05).No definition of appetite level was reported.	No validation measures were described in this article, but the observed test meal used was nearly identical to Dossa et al. (23, 25). Validations in those studies are described above and authors reference Dossa et al. (25) validation.
Mda et al. (29)	Cross-sectional study to compare duration of hospitalization, appetite, and nutritional status of hospitalized HIV-infected and HIV-uninfected children with diarrhea or pneumonia.	South Africa. A total of 189 children aged 2–24 mo admitted to hospital with diarrhea and/or pneumonia formed the overall study, of whom 48 participated in the appetite test. HIV status was determined for children upon admission.Mean ± SD HAZ was −0.67 ± 1.24 for all HIV-uninfected children and −1.68 ± 0.86 for HIV-infected children. Mean ± SD WHZ was −0.60 ± 1.11 for all HIV-uninfected children and −0.67 ± 0.90 for HIV-infected children.Exclusion criteria were receiving vitamin or mineral supplements in the past 2 mo, having a diarrheal or pneumonia episode >72 h on admission, pneumonia and respiratory failure (e.g., hypoxia on supplemental oxygen), and having both diarrhea and pneumonia. Children who were receiving antiretroviral drugs were also not eligible.	Observed feeding of a breakfast test meal as per Dossa et al. (25) and Mda et al. (28). Caregivers of children aged >6 mo who were currently consuming solid foods were asked to participate. Test food was as per Mda et al. (28). Assessment was in clinical setting and repeated on 3 nonconsecutive days over a 2-wk period.The amount of test food consumed (including per body weight), feeding duration, and eating rate were calculated and compared between HIV-infected and -uninfected children. HIV-infected children had longer mean ± SD eating duration (15.6 ± 3.0 min vs. 13.4 ± 3.9 min; *P* < 0.05); lower test food consumption (151 ± 52 vs. 208 ± 74 g; *P* < 0.01); lower test food consumption/kilogram of body weight (18.6 ± 5.8 vs. 25.2 ± 7.4 g; *P* < 0.01); and a slower eating rate than HIV uninfected children (10.1 ± 3.7 vs. 17.6 ± 6.2 g/min; *P* < 0.01). Among children with pneumonia, HIV-uninfected children consumed more test food (*P* < 0.01), in a shorter interval (*P* < 0.01), and at a faster pace (*P* < 0.01) than HIV-infected children. Among children with diarrhea, HIV-uninfected children had greater food consumption (*P* < 0.05), greater amount of food eaten per kilogram of body weight (*P* < 0.01), and a faster eating rate (*P* < 0.01).No definition of appetite level was reported.	No validation measures were described in this article but the observed test meal used was nearly identical to Dossa et al. (23, 25). Validations in those studies are described above.
Nti and Lartey (30)	Observational study to assess the role of caregiver-feeding behaviors on child nutritional status using positive deviant (appropriate weight, nonstunted) and negative deviant (underweight, stunted) children.	Ghana. A total of 100 children aged 6–12 mo. “Positive deviant” children (*n* = 34) had a mean ± SD WAZ of −0.76 ± 0.50 and LAZ of −0.31 ± 0.40; “negative deviant” children (*n* = 66) had a mean ± SD WAZ of −1.85 ± 1.10 and LAZ of −1.52 ± 0.80Children were eligible if they and their mothers were available over the 6-mo study.No health-related inclusion or exclusion criteria were reported.	Home-based assessment of appetite measured the child's interest in food by trained fieldworkers during observed feedings, and classified as whether the child was “highly interested,” “disinterested,” or “refused to eat.” Children received 1 behavioral observation visit/mo, for a period of 6 mo. Caregivers were asked to report children's daily meal frequency during home visits. The period of meal frequency and question used to assess meal frequency were not described.Children who were “highly interested” in food were considered to have good appetite, whereas those who were “disinterested” or who “refused to eat” were considered to have poor appetite.The percentage of time a particular behavior was observed was calculated for each child. Among “positive deviant” children, 86% showed “interest in food” compared with 59% of “negative deviant” children (*P* = 0.025). Throughout the study, 11% and 3%, respectively, of the positive deviants showed either “disinterest in food” during feeding or “refused to eat” as compared with 24% and 17% of negative deviants, respectively (*P* = 0.025). Meal frequency was higher among positive deviant children (3.1 ± 0.4 vs. 2.4 ± 0.6; *P* = 0.001).	NA
Oelofse (31)	Cross-sectional study to assess appetite of 6-mo-old children.	South Africa. Fifty infants aged 6 mo were randomly selected from all mothers visiting a clinic with their infants in Western Cape, South Africa. Of the 50, complete records for 39 were available for analysis.The mean ± SD HAZ was −0.63 ± 1.14 and WHZ was 1.36 ± 1.08.No health-related inclusion or exclusion criteria were reported. Micronutrient-deficiency status was apparently determined postenrollment.	Two relevant assessments:1. Observed feeding of a test meal as per Dossa et al. (23) and Cohen et al. (22). Conducted an ad libitum consumption test of a “common infant cereal” at the study clinic on 3 nonconsecutive days within a 2-wk period. Porridge was prepared by measuring 30 g dry porridge with 90 mL water.2. Caregivers also completed a dietary recall of foods consumed by the child in the 24 h preceding the test meal. Breast-milk intake was estimated at 750 mL/d.Daily and 3-d average energy intake from the 24-h recall and from the appetite test meal were calculated.The average energy intake from the observed test meal over 3 d was 312 ± 182 kJ, which contributed an average of 10% to the average daily energy intake. There was a nonsignificant “within-subject day effect” for the appetite test and a significant “between-subject effect” (*P* < 0.001). Breastfed infants had significantly higher average daily energy intake compared with nonbreastfed infants as measured by the 24-h recall (3584.3 ± 459.9 vs. 2784.1 ± 848.7 kJ; *P* < 0.05), but not from the intake of the test meal (breastfed = 341.6 ± 186.2 vs. nonbreastfed = 259.9 ± 174.6 kJ; *P* value not reported).Additional associations between the observed test meal and serum retinol, iron, and zinc concentrations were all nonsignificant. Cross-sectional associations between child weight, length, knee-heel length, and energy intake from the observed test meal were all nonsignificant.No definition of appetite level was reported.	Validation process involved calculation of Pearson correlation coefficient between the average energy intake from the observed test meal and the average daily energy intake as measured by the 24-h recall. The association was nonsignificant.
Patel et al. (32)	Prospective observational study to evaluate the feasibility and effectiveness of home-based rehabilitation of severely malnourished children	India. A total of 34 severely malnourished children [defined by authors as <70% of the WHO Multicenter Growth Reference Study (33) WHZ median] aged 6–60 mo identified from presentation to a pediatric outpatient department or emergency hospital in a tertiary care hospital in Delhi. All children without complications and with preserved appetite were directly eligible for home-based rehabilitation. Those with complications or loss of appetite were admitted to the hospital. After discharge, they were managed at home using home-based diets.Children with diseases that cause malnutrition such as cerebral palsy, congenital heart disorders, hemolytic anemia, malignancies, metabolic disorders, malabsorption syndromes, chromosomal malformations, or chronic renal and hepatic disorders were excluded.	Hospital-based assessment. Appetite was measured as a criterion for discharge from hospital and for home-based rehabilitation, and was classified as healthy if children “easily consumed” >80% of recommended F-75 feedings during inpatient stay.No further details regarding the assessment of appetite were reported.A total of 29 of the 34 (85%) children qualified for home-based management.	NA
Velásquez et al. (34)	Prospective longitudinal study to evaluate changes in CRP and proinflammatory cytokines in severely malnourished children, before nutritional intervention and upon restoring appetite	Colombia. Twenty severely malnourished children <5 y old with or without apparent infection and with or without anemia. Mean WHZ = −1.3 among children with kwashiorkor (*n* = 10); −3.2 among children with marasmus (*n* = 10); HAZ = −2.6 among children with kwashiorkor; −2.9 among children with marasmus.Children with anemia requiring transfusion, severe dehydration, or edema secondary to kidney, heart, liver, or endocrine diseases were excluded.	Hospital-based assessment. Daily physician evaluation of the infection course, appetite, and weight of children. Feedings of 75 kcal/100 mL of F-75 therapeutic milk were administered every 2 h.Appetite was classified as restored and children were considered “stabilized” when they were able to consume >130 mL ⋅ kg^−1^ ⋅ d^−1^ for 2 d. After appetite restoration, measures of transferrin, ferritin, ceruplasmin, CRP, and cytokines before supplementation with iron.Authors reported that “stabilizing the patients” and “regaining appetite” occurred at about day 5 of treatment with F-75. After appetite restoration, both groups showed a significant decrease in CRP and increases in serum albumin, transferrin, and ceruplasmin. Compared with preappetite restoration, when serum albumin, transferrin, and ceruplasmin values were in “deficiency,” these protein concentrations increased to “normal reference values.”	NA

^1^ART, antiretroviral therapy; CEBQ, Child Eating Behavior Questionnaire; CRP, C-reactive protein; HAZ, height-for-age *z* score; LAZ, length-for-age *z* score; MVMM, multivitamin-multimineral; NA, not applicable; RCT, randomized controlled trial; RUTF, ready-to-use therapeutic food; WAZ, weight-for-age *z* score; WHZ, weight-for-height *z* score.

**TABLE 3:** Evidence table: tools and measures that assessed child appetite through caregiver perception^1^

Study (reference)	Study design and objective	Setting, sample size, and population	Method of appetite assessment	Methods of validation
Tools and measures that assess single appetite domains				
Alarcon et al. (35)	RCT to determine the effectiveness of nutritional counseling with and without Pediasure (Abbott Laboratories) supplementation in improving the growth of children with WHZ <25th percentile and picky eating behaviors.	The Philippines and Taiwan. A total of 92 children aged 36–60 mo with a clinical judgement of “picky eating” defined by authors as refusal of all or certain types of food and <25% for WHZ.WAZ percentile ± SD was 9.6 ± 9.2 for treatment group and 6.9 ± 6.9 for control; HAZ percentile was 19.9 ± 22.2 for treatment group and 15.3 ± 17.5 for control.Exclusion criteria were any acute or chronic infections; fever; allergy to cow milk or other ingredients in the nutritional supplement; iron deficiency anemia or receiving iron therapy; any metabolic, malabsorption, renal, hepatic, cardiovascular, or pancreatic disease; infantile anorexia; or developmental disability.	Parents were asked, in a clinical setting, to rate their child's appetite on a scale of 0–10; “0” represented “ate very little” and “10” represented “ate very much.” Between-group comparisons assessed the change from baseline and 30, 60, and 90 d.Baseline appetite levels were higher in the intervention group compared with controls (4.8 ± 1.8 vs. 4.0 ± 2.1; *P* = 0.023). Appetite levels increased from baseline at each visit (30, 60, and 90 d) for both intervention and control groups, but between-group differences were nonsignificant (numeric results not reported).No cutoff for “normal” level of appetite was reported.	N/A
Arsenault et al. (36)	Community-based RCT to evaluate the effects of zinc in a liquid supplement or in a fortified porridge on growth, dietary intake, appetite, body composition, and hormonal regulators of energy balance.	Peru. A total of 360 children aged 6–8 mo with LAZ <–0.5 were recruited at baseline. Inclusion criteria also included: hemoglobin >80 g/L, no congenital or chronic conditions affecting growth, no use of infant formula (providing >1 mg Zn/d, ≥5 times/wk), and planning to live in the study community for the next 7 mo.	Appetite assessment question based on Brown et al. (20). Investigators visited parents in their homes and asked whether their child's appetite was *1*) usual, *2*) somewhat diminished, or *3*) very diminished on the current day and each day that elapsed from the previous study visit. The percentage of days with diminished appetite was calculated by dividing the number of reported “somewhat” or “very diminished” days of appetite by the number of days of assessment for each child. During pre- and postintervention periods, compared “days of diminished appetite” on days during illness to those without illness. Assessment was conducted over 1–2 d before the intervention and 2–3 mo after the start of the intervention.Responses of “somewhat diminished” or “very diminished” were combined into one category of “diminished appetite.”Caregivers reported that children's appetites were diminished on 19% of days.Groups did not differ in the prevalence of reported diminished appetite either before or during the intervention. The prevalence of diminished appetite before the intervention was a strong predictor of diminished appetite during the intervention (*P* < 0.001). The prevalence of diminished appetite on days with diarrhea or fever (49.3% ± 20%) was significantly greater than on days without fever or diarrhea (14.1% ± 12.3%) (*P* < 0.0001).	Although not reported as validation, the authors did compare reported appetite with energy intake, which was assessed from a 12-h direct observation in children's homes consisting of weighing all food items, recipe ingredients, and beverages served to the child and any uneaten. Breast-milk intake was also measured by weighing the infant before and after every breast-milk feeding using an infant scale.Among children with ≥1 d of reportedly diminished appetite during a day when intakes were recorded, energy intake with diminished appetite was 10% less than when appetite was reported as normal (*P* < 0.0001).
Brown et al. (20)	Cohort study to assess the validity of maternal reports of poor infant appetite.	Peru. Measured caregiver report anorexia (*n* = 153) and energy consumption (*n* = 131) of singleton newborns weighing ≥2500 g at birth living in an economically disadvantaged periurban community outside of Lima. Children followed for 1 y, for 1621 d of child observations.No exclusion criteria or baseline morbidity data were reported.	Mothers were visited in their homes and asked to rate their child's appetite as “less than usual,” “same as usual,” or “greater than usual” 3 times weekly for 52 wk. Anorexia was classified when the child's appetite was reported as “less than usual” on a particular day. New episodes of anorexia were assessed for only when the infant's appetite was reportedly normal (“same as usual”) or increased (“greater than usual”) for an interval of ≥2 d before the next assessment.Mothers reported that their infants had reduced appetites on ∼15% of total reported days. The prevalence of poor appetite increased with child age, from 22 of 1000 d among infants aged <1 mo to 313 of 1000 d of observation among children aged 11 mo. The incidence of new episodes of anorexia increased from 8 episodes/1000 d at risk in month 1 to 63.4 episodes/1000 d at risk at 11 mo of age. Also assessed duration of anorexia in days (median = 3.0 d among infants aged <6 mo and 4.5 d among infants aged ≥6 mo). Older infant age and the presence of fever, diarrhea, and respiratory illnesses were each associated with reduced appetite; however, less than one-third of new episodes of anorexia were associated with symptoms of illness.See Table 2 for details of food intake assessment.	Reported anorexia was validated against observed dietary intake.Mean total energy intakes on days with reported anorexia were 338 ± 88 kJ/kg body weight in infants 1–6 mo old and 299 ± 92 kJ/kg body weight in infants aged >6 mo, compared with 395 ± 92 and 342 ± 88 kJ/kg among children whose appetites were reportedly normal in the same age group (*P* < 0.001 adjusted for age within age categories, body weight. and illness symptoms).Adjusted intraindividual total energy intakes were nearly 15% less on days with reported anorexia (*P* < 0.001).The authors concluded, “Thus, the mothers’ reports of anorexia did indeed coincide with observed decreases in their infants’ energy consumption…” and were more accurate for nonbreast-milk sources than breast-milk sources.For further details of this validation see footnote 2.
Ciliberto et al. (21)	Case series to determine the safety and effectiveness of outpatient management of edematous malnutrition.	Malawi. A total of 219 children aged 1–4 y with mild edematous malnutrition (<0.5 cm pitting edema on the dorsum of the foot) and “good appetite.” Mean ± SD baseline HAZ was −2.9 ± 1.4; mean WHZ was −1.7 ± 0.9.	Caregiver was asked, in a clinical setting, if the child was consuming food when it was offered. If caregivers responded “no,” they were asked to estimate the proportion of food normally consumed by the child.No results regarding caregiver perception of appetite were reported.Food intake was also assessed (see Table 2). Caretaker report of food consumption and dietary intake quantification were not compared.	N/A
		Children were drawn from 2 large previously reported community-based malnutrition studies. Children aged 12–60 mo who presented to 1 of 7 nutritional rehabilitation units or 1 of 8 villages in a community-based prevention trial were screened. No additional health/morbidity inclusion/exclusion criteria were noted.		
Cohen et al. (22)	RCT to assess food acceptance based on timing of solid food introduction. Women were visited weekly during the first 4 mo postpartum. At week 16, they were randomly assigned to exclusive breastfeeding until 26 wk, solid foods with ad libitum breastfeeding, or solid foods plus breastfeeding as often as before.	Honduras. A total of 141 primiparous, low-income mothers aged ≥16 y willing to exclusively breastfeed for 26 wk, not employed outside the home, and living in conditions of poor environmental sanitation. Infants were healthy, full-term, and with birth weight ≥2000 g.	Home-based interviews with mothers during 9- and 12-mo visits about usual food acceptance of 20 common foods and rated on the following scale: 1 = “eats well,” 2 = “accepts,” 3 = “difficult to get to eat,” 4 = “refuses.”Mean ± SD food acceptance scores were similar among intervention groups: the overall mean at 9 mo was 1.3 ± 0.2 and 2.0 ± 0.2 at 12 mo, indicating that infants accepted most foods well. All 20 foods were accepted equally well among the groups, except liver and bell peppers. By 12 mo, acceptance of even these foods no longer differed between groups.Food intake was also assessed (see Table 2). Maternal report of food acceptance and dietary intake quantification were not compared.	N/A
Dossa et al. (23)	RCT to assess whether a combination of MVMM supplement and additional iron treatment improved the appetite, morbidity status, hemoglobin, and growth of children.	Benin. A total of 154 children aged 18–30 mo who were stunted (HAZ <−2) and anemic (hemoglobin <110 g/L) were randomly assigned to 1 of 4 arms (4 children lost to follow-up): MVMM/Fe (*n* = 39); MVMM/placebo (*n* = 38); placebo/placebo (*n* = 39); or placebo/Fe (*n* = 37). Mean HAZ for each group was: MVMM/Fe = −2.67 ± 0.66; MVMM/placebo = −2.76 ± 0.85; placebo/placebo = −2.45 ± 0.60; placebo/Fe = −2.66 ± 0.59. Overall mean HAZ for total study sample at baseline was −2.6 ± 0.7.	Mothers were asked to report on child's appetite before test day responding to study question: “How did your child eat throughout the day before the test day?” Response options were either: 1 = “my child ate well,” or 2 = “my child did not eat well.” The setting in which the questionnaire was administered was not specified.Children who were reported by their mothers to have good appetites/eat well had higher energy intake from the test food compared with their counterparts who did not have good appetites (*P* < 0.05) before and after supplementation.No difference was found between groups in changes in maternal report of appetite over the 6-wk supplementation period.Food intake was also assessed (see Table 2). Caretaker report of appetite and dietary intake quantification were not compared.	N/A
		Among 272 screened children with HAZ <−2, 4 were excluded due to an HAZ <−5, and 22 were excluded because they did not eat rice or did not like rice, according to mothers. Of the remaining 246, 154 were found to be anemic and were randomly assigned.		
Dossa et al. (26)	RCT to assess effect of daily micronutrient supplementation on appetite and growth among stunted children. Anthropometric, appetite, and morbidity status were assessed weekly through 6 wk postintervention.	Benin. A total of 101 stunted (HAZ <−2) children aged 17–32 mo (mean ± SD HAZ = −2.8 ± 0.7) randomly assigned to MVMM treatment (*n* = 48) or placebo (*n* = 53) for 6 wk. All participants needed to accept the fermented maize porridge used for control feeding.No health-related inclusion or exclusion criteria were reported.	Maternal perception of child appetite was assessed using the same question as the Dossa et al. (23) study. The setting in which the questionnaire was administered was not specified.The percentage of days of reported good appetite increased from before to after (6 wk) supplementation in both the MVMM group (53% to 73%; *P* < 0.05) and the placebo group (54% to 72%; *P* < 0.05).Food intake was also assessed (see Table 2). Maternal report of appetite and dietary intake quantification were not compared.	N/A
Huynh et al. (37)	Cohort study to assess the impact of dietary counselling and long-term macro- and micronutrient supplementation over 48 wk on ponderal and linear growth patterns and related health aspects.	Philippines. A total of 200 healthy children aged 36–48 mo and “at risk for undernutrition” defined as WHZ between 5th and 25th percentile. Parents received 3 sessions of dietary counselling at baseline and 4- and 8-wk visits. Children received 2 servings of food supplementation/d for 48 wk.	Parents were asked, in a clinical setting, to rate their child's appetite over the previous 24 h. A visual analog scale was used to assess appetite on a 1–10 scale (1 = “ate very little” to 10 = “ate very much” on the previous day). The authors reported the mean group score at baseline and 4, 8, 16, 24, 32, 40, and 48 wk. Compared with baseline (mean = 6.4), appetite scores were higher for all postbaseline tests (*P* < 0.0001), with the highest mean score (9.0) at 48 wk. Increases in appetite corresponded with increases in reported physical activity levels and decreased reported sick days, as well as increases in energy intake and WHZ, and HAZ percentiles.	N/A
		Exclusion criteria were a history of preterm delivery, birth weight <2500 or >4000 g, or current chronic infections (except for intestinal parasites), diarrhea, acute and chronic hepatitis B or C, HIV or tuberculosis, congenital or genetic disorder, or infantile anorexia nervosa.	No cutoff for “normal” level of appetite was reported.	
Namdari et al. (38)	Cross-sectional study to assess the association between serum folate concentrations and appetite status of children.	Iran. A total of 127 healthy children aged 36–72 mo (mean ± SD age: 58.3 ± 12.1 mo) randomly selected from 20 preschools in Tehran. Mean ± SD BMI (kg/m^2^) was 15.3 ± 2.2.Eligibility for inclusion was based on being healthy according to medical history and physician examination. Exclusion criteria were having acute intestinal infections, acute respiratory failure, chronic renal failure, active liver disease, hemolysis of RBCs, or fever.	Mothers were asked to respond to “How do you describe the amount of food that normally has been eaten by your child in the last few days?” Using a 1- to 21-point scale that corresponded to “very little,” “little,” “average,” “much,” or “very much,” mothers were asked to circle the number that indicated their child's appetite during the last few days. The setting in which the questionnaire was administered was not specified.No cutoff for “normal” level of appetite was reported.The relation between serum folic acid and appetite was evaluated by multivariable linear regression modeling with the appetite score on a 21-point rating scale as the main dependent variable. Regression models showed a positive association of serum folic acid concentrations on children's appetite score (β = 0.29; 95% CI: 0.23, 0.36). BMI (β = 0.82; 95% CI: 0.73, 0.90), and having a mother who did not work outside the home (β = 0.69; 95% CI: 0.31, 1.06) and socioeconomic status (measurement not specified) (β = 0.42; 95% CI: 0.06, 0.78) were also independently positively associated with higher appetite scores.	N/A
Ruel et al. (39)	RCT to measure the effect of zinc supplementation on morbidity from diarrhea and respiratory infections.	Guatemala. A total of 89 children aged 6–9 mo assigned to treatment (*n* = 45) or placebo (*n* = 44); 56% of children were stunted (LAZ <−2) at baseline, and none (0%) were wasted (WLZ <−2).No health-related inclusion or exclusion criteria were reported.	Mothers were visited in their homes and asked about the loss of appetite, or anorexia, among other variables in a daily morbidity assessment. Classification of anorexia was based on the mother's definition.Children from the zinc-supplemented group had lower reported days (25th–75th percentile] of anorexia (4.7; 1.9–6.8) than those from the placebo group (6.1; 2.7–7.8), although this difference was not significant. Multivariable regression models confirmed lack of association of the intervention with anorexia (as well as respiratory and febrile illness prevalence, although an effect on reduction in diarrheal illness was found, especially among children with lower WLZ at baseline).	N/A
Sawadogo et al. (40)	Cohort study to assess the impact of common diseases (diarrhea, acute respiratory infections, and febrile illnesses) on the nutritional status of children.	Burkina Faso. A total of 114 children from 30 villages of the same rural province aged 6 mo ± 15 d, followed for 18 mo. At baseline, mean ± SD LAZ was −0.96 ± 0.93; mean WLZ was −0.18 ± 0.96; 10.5% had LAZ <−2; 2.6% had WLZ <−2.All children living in 30 selected villages were eligible if caregivers agreed and intended to stay in the area for the 18-mo study period. No health-related inclusion or exclusion criteria were reported.	Mothers reported whether the observed illness had resulted in the child losing his/her appetite. Information concerning appetite was only retained if it was connected with a specific illness and not due to other causes such as a monotonous or unbalanced diet. An “appetite coefficient” was used to calculate an “overall morbidity score,” which took into account both the duration of the illness and its effect on the child's appetite. The “overall morbidity score” was calculated as ln(1 + D)A, where D is the duration of the illness in days, A is the appetite coefficient = 2 (loss of appetite), 1 (no loss of appetite), or 0 (no illness). The setting in which mothers were asked about appetite and morbidity was not specified.At time T, children with higher morbidity scores had WLZ scores 0.34 lower than healthy children (*P* < 0.0001). At time T + 1, the children with higher morbidity scores had a lower mean LAZ than children with lower morbidity scores (LAZ difference = −0.09; *P* = 0.02).Using only appetite coefficient scores, children with higher appetite scores (worse appetite) had WLZ scores 0.34 lower than healthy children (*P* < 0.0001) at time T. At time T + 1, the children with higher appetite scores (worse appetite) had a nonsignificantly lower mean LAZ than children with lower morbidity scores (LAZ difference = −0.08; *P* = 0.02).	N/A
Stoltzfus et al. (41)	RCT to assess effects of low-dose iron supplementation and/or anthelminthic treatment (MEB) on growth, anemia, and appetite.	Tanzania. A total of 614 children aged 6–59 mo without severe anemia. Stunting occurred in 38% of children and 31.2% were underweight. Wasting occurred in 3.8% of children.Children with severe anemia (<70 g/L) were excluded and treated. This was the sole exclusion criterion.	Assessment based on Brown et al. (20). Mothers were asked, in a clinical setting and in the local language, “Lately, how has your child's appetite been?” Response options were on a 1- to 5-point scale from “very bad” to “very good.” At the 12-mo follow-up visit, 84% identified their child's appetite as “very good.” Therefore, the other 4 categories were grouped together to create the variable “poor appetite.”Both interventions were associated with reduced maternally reported poor appetite by ∼40% in crude models and 50% in adjusted models, with a consistent effect across age groups: AOR (95% CI) of poor appetite for Fe treatment = 0.51 (0.27, 0.95); AOR for poor appetite for MEB treatment = 0.52 (0.30, 0.89).The intervention had no significant effect on anemia outcomes in all study groups. Iron supplementation had no significant effects on either mild wasting (WLZ <−1) or stunting (LAZ <−2). MEB treatment was protective against mild wasting in younger children (AOR = 0.38; 95% CI = 0.16, 0.90), but was associated with mild wasting in children >48 mo (AOR = 2.88; 95% CI = 0.82, 10.14).	Appetite question was previously validated by Brown et al. (20), where maternal report of anorexia (appetite less than normal) was significantly positively associated with actual intake, infant age, and illness events. Stoltzfus et al. (41) noted that they did not attempt to validate the assessment question in their study context, and that the distribution of responses indicated that mothers did not fully appreciate the meaning of the 5-point Likert scale. Authors suggested that this could be due to mothers being “averse to reporting anything that is ‘bad’ about their children” and recommended that future studies of appetite in this setting will require “more informative methods” to be developed.
Tools and measures that assess multiple appetite domains				
Hatamizadeh et al. (42)	RCT to assess the impact of folic acid supplementation on children's weight gain and appetite.	Iran. A total of 61 children aged 36–60 mo who were determined to be healthy according to medical examination and with WAZ <25th percentile and WHZ <25th percentile were randomly assigned to receive folic acid (*n* = 31) or placebo (*n* = 30) for 20 d.Eligibility criterion for inclusion in the RCT was a score of ≤10 in question 1 (general appetite assessment) of the questionnaire.	Question 1 (a general appetite assessment) was used to determine eligibility for the study and asked parents to score children's appetite using a 1- to 21-point Likert score (very poor to vigorous).Questions 2–5 assessed 4 components of appetite: *1*) rate of eating (“eats very slow” to “eats very fast”); *2*) proportion of food consumed (“hands out full plate” to “empties dish entirely”); *3*) range of places in which child will eat (“eats only in a special setting” to “eats anywhere”); and *4*) the amount of food generally consumed (“eats a little” to “eats very much”). Each of these 4 components was measured by a single question on a 1- to 5-point scale, to create a 4- to 20-point scale. The authors initially tested a fifth “appetite component” that measured the “variety of foods a child accepts”; however, this item was eliminated due to lack of significance with the general appetite assessment question. Questions 2–5 were assessed at baseline, during the intervention (day 20), and 40-d postintervention (day 60).	Authors describe a validation process comparing results of question 1 to questions 2–5 (4 components of appetite) among a subset of 30 children. The authors dropped a question/domain regarding food variety from the final tool because the Spearman correlation coefficient with question 1 was nonsignificant. The final reported Cronbach's α (a measure of internal consistency) of the 4 appetite components (questions 2–5) was 0.72.
			A final question (question 6) assessed the perceived change in appetite in response to the syrup consumption (response options ranged from 1 = “got worse” to 7 = “increased vigorously”). In the final assessment (day 60, 40 d postintervention), the response options to this question were simplified to 1 = “got worse”; 2 = “no change”; 3 = “got better”. Question 6 was assessed during the intervention (day 20) and 40 d postsupplementation (day 60).The setting in which the questionnaire was administered was not specified.Poor appetite was defined as a score of ≤10/21 on question 1.Between baseline and day 20 of supplementation, the mean difference in appetite scores measured according to the difference in sum of questions 2–5 between groups was higher in the folic acid vs. the placebo group (mean difference: 1.7; 95% CI: 0.1, 3.4; *P* = 0.04); however, this difference was not maintained at 40 d. Based on responses to question 6, 68% of children in the folic acid group had reportedly improved appetite after consuming the syrup, compared with 40% of placebo children at 20 d of supplementation. At 40 d postsupplementation, 27% of folic acid children had reportedly improved appetite compared with 38% of placebo children. Expressed as an RR, the likelihood of increased appetite in response to the folic acid supplementation at 20 d of supplementation was higher in the treatment group than in the placebo group: RR = 1.7 (95% CI: 0.3, 2.8; *P* = 0.03); however, the difference was not maintained at 40 d postsupplementation.	
Khademian et al. (27)	RCT to assess effect of daily zinc administered for 12 wk on appetite and its subscales.	Iran. A total of 96 children aged 24–74 mo with a chief complaint of anorexia were randomly assigned to receive zinc (*n* = 48) or placebo (*n* = 48); 78 children completed the trial (77 in zinc, 38 in placebo). For inclusion, anorexia could not be from a major organic cause (e.g., anemia, urinary tract infection, parasitic infection, hemoglobin <11 g/dL). Children with gastrointestinal complications in the group receiving zinc were excluded. The nutritional status of children was not reported.	Before the intervention, the CEBQ was administered to caregivers in Persian and evaluated as published elsewhere [Wardle et al. (43)]. The CEBQ consists of 8 domains, each measured using a 5-point scale, ranging from “never” to “always”: Food Responsiveness, Emotional Overeating, Enjoyment of Food, Desire to Drink, Satiety Responsiveness, Slowness in Eating, Emotional Undereating, and Food Fussiness.In addition, total mean calorie, carbohydrate, protein, fat, and zinc intakes were estimated pre-and postsupplementation to assess pre- and postsupplementation domains alongside food consumption (see Table 2). The setting in which the questionnaire was administered was not specified.No definition of appetite level was reported. The method of assessing the complaint of anorexia upon enrollment was also not specified.The authors concluded that zinc supplementation ”could improve calorie intake in children” by noting significantly pre-post changes on CEBQ subscales within groups. Among the supplementation group, Emotional Overeating (*P* = 0.006) and Food Responsiveness (*P* = 0.03) were significantly different between baseline and endline. In the control group, Emotional Undereating was different between baseline and endline (*P* = 0.011). The specific CEBQ subscale scores, the direction of change (increase or decrease) from baseline to endline, and the statistical comparison of intervention to control groups were not reported.Food intake was also assessed (see Table 2).	N/A. Used a previously validated tool (CEBQ) that was initially intended to be used for overeating and obesity risk. In the Iranian context, it was applied to children who suffered from anorexia. The authors describe the validation of the questionnaire as a supervised translation process into Persian. No additional assessment of the tool in relation to another measure of appetite or dietary intake was described.
Najib et al. (44)	RCT to assess the effect of CH (an appetite stimulating histamine antagonist) on ponderal and linear growth and BMI in children with mild to moderate undernutrition.	Iran. A total of 82 children aged 24–64 mo with mild to moderate undernutrition, defined as 75–90% and 60–74% of standard weight, respectively, according to the Gómez classification. Randomly assigned to receive CH + MV (*n* = 41), or MV only (*n* = 41) for 4 wk. Anthropometrics were measured at baseline, 4 wk after intervention, and 4 weeks after discontinuation. Patients with a history of antihistamine intolerance, or receiving sedatives, narcotics, steroids, or appetite stimulants within 1 mo before enrollment, or with comorbidities such as urinary tract infections, metabolic disturbances, chronic renal failure, and cystic fibrosis, were excluded.	Evaluated 4 domains in a clinic-based setting: “willingness to eat,” “unwillingness to eat after a few tablespoons of food,” “attention to eating,” and “mean number of meals.” The response options for these questions are not reported. Values were assessed pre- and postintervention and compared between study groups. After 4 wk of CH therapy, unwillingness to eat after a few tablespoons of food was lower in the CH group than in the controls (10.0% vs. 32.4%; *P* = 0.02). Attention to eating was higher in the CH group after intervention (75.0% vs. 48.6%; *P* = 0.02). The CH group had a higher increase in the mean ± SD number of meals after the intervention compared with the placebo group (4.0 ± 0.8 vs. 3.7 ± 0.8; *P* = 0.04). The questions used to assess the 4 domains of appetite were not described.No definition of appetite level was reported.	N/A
Umeta et al. (45)	RCT to investigate whether zinc supplementation could improve the low rate of linear growth of healthy breastfed infants.	Ethiopia. A total of 200 healthy breastfed infants aged 6–12 mo; 100 nonstunted (LAZ >−2) infants were matched for age and sex with 100 randomly selected stunted (<–2) infants and then randomly assigned to zinc or placebo. Among stunted children, the mean LAZ was −2.7 in the zinc and −2.8 in the placebo groups. Among nonstunted children, the mean LAZ was −0.7 in the zinc and −0.6 in the placebo groups.Infants needed to be breastfed, apparently healthy based on visual assessment, and free from intestinal parasites.	Daily home-based assessment of “anorexia incidence” by field assistants who asked mothers “whether the child refused to breastfeed, whether the frequency, duration or intensity of breastfeeding was reduced, or whether the frequency or amount of weaning foods consumed was reduced.” No further details on this appetite/anorexia assessment were reported. Differences in growth between groups was the primary outcome; differences in anorexia and morbidity between groups were also calculated.Zinc supplementation was significantly associated with reduced incidence of anorexia among stunted (3 episodes in the intervention vs. 15 in the placebo group) and nonstunted children (0 episodes in the intervention vs. 4 in the placebo group) (*P* < 0.05). The authors found significant ponderal and length gains in the intervention vs. placebo group, especially among the stunted children. Children in the intervention group experienced lower morbidity (cough, diarrhea, vomiting, fever) incidence compared with the placebo group in stunted, but not in nonstunted, children. The authors concluded that the improvements in growth could be due, at least in part, to an effect on appetite and reduced illness from zinc supplementation.An episode of anorexia was defined based on a refusal to breastfeed; reduced frequency, duration, or intensity of breastfeeding; or reduced frequency or amount of weaning foods.	N/A

^1^AOR, adjusted OR; CEBQ, Child Eating Behavior Questionnaire; CH, cyproheptadine hydrochloride; HAZ, height-for-age *z* score; LAZ, length-for-age *z* score; MEB, mebendazole; MV, multivitamin; MVMM, multivitamin-multimineral; N/A, not applicable; RCT, randomized controlled trial; WAZ, weight-for-age *z* score; WHZ, weight-for-height *z* score; WLZ, weight-for-length *z* score.

^2^When breast-milk energy consumption was examined separately in relation to the maternal appetite reports, intakes declined by only ∼5% on days with reported anorexia in infants <6 mo of age and no change in consumption among older children. The authors reported no significant differences in breastfeeding frequency or duration on days with or without reported anorexia, regardless of the exclusivity of breastfeeding or age group. In contrast, energy intake from nonbreast-milk sources was 25–35% less in both age groups on the days of reported anorexia. The authors concluded that mothers more accurately diagnosed poor appetite in relation to nonbreast-milk energy sources. The authors also sought to determine factors associated with mothers’ ability to predict decrements of ≥42 kJ/kg and found no significant differences in characteristics assessed, including maternal education, age of closest sibling to the index child, or socioeconomic status (based on housing quality). However, mothers who diagnosed anorexia “correctly” using this definition fed their infants a lower proportion of total energy as breast milk than those whose diagnosis of poor appetite did not correspond with the indicated decrement in intake (60.7% ± 29.1% vs. 73.3% ± 26.6% of intake, respectively; *P* = 0.033). As part of the validity assessment of maternally reported anorexia, the adjusted odds [adjusted for age (within age group), sex, presence of stunting, and season of year] were calculated for the presence of fever, diarrhea, and respiratory illness, as well as breast-milk and nonbreast-milk feedings. Fever and diarrhea were both significantly associated with increased odds of reported anorexia across all age strata (OR for fever ranged from 2.4 to 3.1 across 4 age strata and 1.7 to 2.1 for diarrhea), whereas respiratory illness was only significantly associated among older infants (6–8 mo and 9–11 mo), and point estimates were smaller (OR: ∼1.3). Breastfeeding was significantly inversely associated with reported anorexia, but only among those aged <6 mo (OR: 0.2–0.3). Other types of feedings (nonhuman milk and solid food) were assessed among <6-mo-olds only, because older infants were uniformly not exclusively breastfed. Only the consumption of nonhuman milk was significantly associated with reported anorexia among 2- to 5-mo-olds. Solid milk was inversely associated with reported anorexia among <2-mo-olds. The authors did not report a multivariable model that includes all of the food-consumption variables simultaneously to assess for independent association.

The 23 included studies were conducted in 15 distinct countries in sub-Saharan Africa (*n* = 11), the Middle East (*n* = 4), South Asia (*n* = 1), Southeast Asia (*n* = 2), and Latin America (*n* = 5). Nearly half of the studies (*n* = 11) were among children with normal nutritional status, whereas 5 were among children with moderate or severe wasting and 6 were among those with stunting or included study populations with a high prevalence of stunting. Nutritional status was not reported in one study (27). The setting where the appetite assessment was conducted varied from children's homes (*n* = 6), a field office (*n* = 6), or in a health facility (*n* = 7), and was not specified in 4 articles. The purpose for the appetite assessment varied substantially across the studies; 14 included this measurement in response to behavioral, dietary, or pharmacologic interventions, whereas 9 compared appetite in relation to micronutrient, nutritional, morbidity status, or other assessments of appetite (e.g., comparing an observed feeding of a test meal to energy intake on the basis of a 3-d diet diary).

### Tools and measures that assessed child appetite through direct observation of dietary intake or quantification of intake based on caregiver record

Children's appetite was measured by direct observation of actual intake (*n* = 8), caregiver-recorded logs or 24-h recall (*n* = 1), or both (*n* = 3) with subsequent calculation of food or energy intake. In addition, we included one study in this category that involved trained fieldworkers directly observing caregivers feeding their children; however, instead of quantifying intake, the fieldworkers were asked to report children's interest in eating during the feeding episode (30). Table 2 summarizes these 13 studies. Only those methods that quantified nutrient intake via caretaker-recorded or -reported intake of foods or that involved study personnel directly observing feedings were included in this category. In other words, methods restricted to caretaker reports of number of daily meals without quantification of actual dietary intake were excluded from this category and are reported in Table 3.

We identified 7 studies that used a method, based on a 1990 study from Kenya, that involves an observed feeding to measure food consumption (22, 23, 25, 26, 28, 29, 31, 46). Five of these 7 studies were from the same author group and were conducted in Benin and South Africa (23, 25, 26, 28, 29). The method involves ad libitum feeding of a breakfast after an overnight fast (23, 25, 26, 28, 29, 31) or an unannounced midday meal at caregivers’ homes (22). The exact test meal used varied across the studies but mostly consisted of a study-specified porridge-type infant food that included the following: *riz-au-gras* (rice cooked in broth) (23, 25), *aklui* (a maize porridge that is more liquid than the *riz-au-gras*) (23, 26), or common infant cereal (28, 29, 31). In some instances, authors described specific recipes for food preparation, such as in South Africa where a honey and wheat infant cereal (Nestlé Nestum no. 2) was prepared as 25 g of dry porridge mixed with 160 mL of 50°C fresh milk (29). The test meal in a Honduran study was not investigator-specified; rather, the researchers quantified intake of home-prepared foods as determined by mothers (22).

The method used by the Dossa et al. (23, 25, 26) and Mda et al. (28, 29) research teams measured food consumption over a series of 2 or 3 feeding episodes in the same meal sitting (i.e., not over multiple days). Researchers measured *1*) the total amount of food consumed in grams during each specific episode and *2*) the rate of eating, by timing the feeding episodes and quantifying in grams per minute, as well as the duration of each feeding episode. In addition, the weight of the remaining food not consumed (measured in grams) and the amount of food per kilogram of body weight were also calculated (23, 25, 26). The Dossa et al. (23, 25) studies estimated a lower but nonsignificant consumption of the test food when ad libitum water consumption was offered. The authors reported that children typically consumed 70–90% of food within the first feeding attempt (25). The within-subject day-to-day variation in the test food intake across 3 feeding episodes was 40% for *aklui* and 25% from *riz-au-gras* (23). Oelofse (31) based his approach on the Dossa et al. (23) method, but assessed only 1 test-feeding episode on 3 nonconsecutive days (31). Another study observed that children left 25–40% of offered food unconsumed during observations of feeding home-prepared meals (22).

One study in our review conducted formative research among mothers on food preferences of young children to develop their test food, noting that this food should be culturally appropriate and well preferred (25). The authors also noted that liquid test food (e.g., maize porridge) is more easily standardized than solid foods, and multiple episodes of feeding within the same meal were necessary to ensure that children were completely satisfied before they finished eating (25).

Although the porridge-based test meal studies described above were largely used among stunted, but nonwasted populations, 3 of the direct observation studies in our review assessed appetite among children with severe acute malnutrition. These studies used F-75 therapeutic milk formula (32, 34) or ready-to-use therapeutic food (RUTF) (21) as test foods in their appetite assessments.

Other studies that quantified nutrient intake to assess appetite included maternal recording of 3 d of food intake for their children in Iran (27). A Peruvian study used direct observation on 1 or 2 d/mo across the first year of life to compare calculated energy intake on the basis of maternal reported anorexia in children (20). Their analysis comparing maternal reported anorexia and nutrient intake, as well as 2 other publications’ analyses with regard to the correlation between the observed test meal results with detailed 3-d diet diaries are described below in the section entitled “Validation of appetite assessment methods and tools” (23, 31).

### Tools and measures that assessed caregiver perception of child appetite

#### Measurement domains of caregiver perception tools

Table 3 summarizes the evidence from the 16 studies that administered questionnaires to caregivers asking them to rate a child's appetite or related domains (e.g., emotional undereating). Twelve of these studies assessed 1 domain of child appetite, either through a single question (*n* = 11) or multiple questions that assessed the same appetite domain (*n* = 1) (22). The majority focused on the child's willingness to consume food or foods (*n* = 2) or his or her current intake compared with usual intake (*n* = 8). The remaining 4 caregiver-perception studies assessed multiple domains ranging from a child's refusal to breastfeed and changes in the frequency, duration, and intensity of breastfeeding (45) to factors such as the rate of eating, the quantity of food ingested, the variety of foods consumed, and the ranges of places a child will eat (42).

#### Measurement methods of single-domain caregiver-perception tools

Among the 12 single-domain caregiver-perception assessments, response options were assessed through questionnaires administered to mothers by research staff. Caregivers were asked to respond with a “yes or no” or other dichotomous reply to a given question (*n* = 3) (21, 23, 26) or were asked to answer with a Likert-type scale (*n* = 6) (20, 22, 35, 36, 38, 41). Of the 3 remaining single-domain studies, one study based in the Philippines used a visual analog scale to facilitate caretaker quantification of their child's appetite on a 10-point scale (37). Another study allowed caregivers to self-define anorexia and was presumably classified as present or not present (47). Finally, one study did not specify how the response options were captured (40).

#### Measurement methods of multiple-domain caregiver-perception tools

Notably, all 4 of the multiple-domain caregiver-perception studies measured appetite in response to an intervention within a trial. An trial based in Iran used its first question to ask caregivers to rate their child's appetite on a 21-point Likert-type scale from “very poor” to “vigorous,” followed by a 4-domain/4-question survey using Likert-type subscales ranging from 1 to 5. The authors’ sixth and final question was the only one used to assess response to the intervention and asked caregivers how appetite changed after folic acid supplementation or placebo with the use of a Likert-type range from 1 = “got worse” to 7 = “increased vigorously” and then simplified to 1 = “got worse”, 2 = “no change”, and 3 = “got better” (42). In a zinc supplementation trial in Ethiopia, caregivers’ perceptions of 6- to 12-mo-olds’ level of “anorexia” were measured by assessing the following: *1*) breastfeeding refusal; *2*) frequency, duration, or intensity of breastfeeding reduction; and *3*) decreased frequency or amount of weaning foods consumed (45). Investigators of another randomized controlled trial from Iran asked parents 4 questions pertaining to “willingness to eat,” “unwillingness to eat after a few tablespoons of food,” “attention to eating,” and “mean number of meals” to assess response to cyproheptadine hydrochloride (an appetite-stimulating histamine antagonist) among undernourished 2- to 5-y-olds (44). Another study used a modified version of the Child Eating Behavior Questionnaire (CEBQ) to generate a score using a 5-point “never” to “always” Likert-type scale (27). Developed for preschool children, the CEBQ consists of 8 subscales: food responsiveness, emotional overeating, enjoyment of food, desire to drink, satiety responsiveness, slowness in eating, emotional undereating, and food fussiness. Although the CEBQ was originally developed to assess food behaviors associated with overeating and obesity risk (48), we retained this instrument in this review because it was adapted for use in an LMIC in a sample of preschool children with reported anorexia and not in relation to overeating.

### Defining a good or healthy appetite

#### Appetite classification from direct feeding observation or nutrient quantification studies based on caregiver report

Among the 13 studies that used direct dietary observation, only 3 described a definition or cutoff for healthy or good or unhealthy or bad appetite. In Malawi, acutely malnourished children were considered to have a good appetite if they consumed 100% of a 30-g portion of RUTF during a 20-min feeding episode (21; M Manary, personal communication, 25 October 2017). In India, Patel et al. (32) classified children as having a healthy appetite, and thus suitable for home-based therapy for severe malnutrition, if they were “easily consuming” >80% of recommended foods during an inpatient stay. In an observational study in Ghana, trained fieldworkers assessed children's eating behaviors and classified children as “highly interested,” “disinterested,” or “refused to eat” on the basis of monthly behavioral observations. Children who were “highly interested” in food were considered to have good appetite, whereas those who were “disinterested” or who “refused to eat” were considered to have poor appetite (30). Although the remaining studies of direct dietary observation did capture the amount of food consumed (in grams), the rate of eating (measured in grams per minute), duration of feeding episodes (measured in minutes), and/or the proportion of food consumed from what was offered, they did not classify results into categories of good and poor appetite. No definition of appetite level was reported for the 2 studies that estimated nutrient quantification on the basis of caregiver report (27, 31).

#### Appetite classification based on caregiver-perception studies

Seven of the 16 caregiver-perception studies had a discernable “cutoff” for classifying poor appetite in children. Among the single-domain studies, 2 studies by Dossa et al. (23, 26) assessed caregiver perceptions and used a dichotomous response option of “good appetite” if the child “ate well” compared with “did not eat well” (23, 26). The single-domain question used within a randomized trial in Iran classified poor appetite as having a score of <10 on a 1–21 scale, whereas their multiple-domain tool did not define good compared with poor appetite (42). Three additional single-domain studies used a question based on that by Brown et al. (20) that classified “reported anorexia” on the basis of Peruvian caregivers’ assessment of appetite as “less than usual” compared with the “same as usual” or “greater than usual” on a particular day (20). When the question was applied in a later study in Peru, the possible appetite response options “somewhat diminished” or “very diminished” were interpreted as “diminished appetite” (in contrast to the response option “usual”). Last, a 5-point scale ranging from “very bad” to “very good” was collapsed with the 4 lowest categories defining “poor appetite” because 84% responded “very good” (41).

 Among the multiple-domain studies, only one explicitly defined anorexia on the basis of refusal to breastfeed; reduced frequency, duration, or intensity of breastfeeding; or reduced frequency or amount of weaning foods consumed (45).

### Application of appetite tools to assess recovery from illness and response to intervention

Several studies evaluated appetite prospectively and specifically in the context of recovery from illness. For example, a longitudinal study based in Colombia assessed C-reactive protein and proinflammatory cytokine changes among severely malnourished children shortly after appetite restoration on the basis of an observed feeding test of therapeutic milk (34). In Burkina Faso, mothers were asked to identify if their child's appetite diminished in connection with a recent illness (40). A “morbidity score” was calculated that took into account both the illness duration and appetite loss resulting from illness, and not due to causes such a monotonous or unbalanced diet (40).

Thirteen intervention studies featured appetite as at least one outcome measure (22, 23, 26, 27, 29, 35–37, 41, 42, 44, 45, 47), of which 5 showed no significant improvement in assessed appetite (22, 23, 26, 36, 47). Several studies measured appetite and growth outcomes and some even described causal frameworks, which included appetite and morbidity reduction in the pathway between micronutrient interventions and improved growth. For example, these outcomes were assessed along with hemoglobin status in an iron and multiple micronutrient supplementation trial among children with stunting and anemia. There were no improvements in appetite, morbidity, growth, or long-term hemoglobin status in relation to the intervention (23).

Our review included 4 zinc-supplementation randomized trials (27, 32, 36, 47), 2 of which assessed growth outcomes in addition to appetite (36, 45). In Iran, daily supplementation with 10 mg for 3 mo among children aged 24–74 mo with a chief complaint of anorexia resulted in improved measures of appetite, including higher caloric intake and improved caregiver perceptions of some domains of appetite (27). In Guatemala, the same dose of zinc was given to infants aged 6–9 mo without an effect on maternal perception of appetite loss (47). Three milligrams of zinc given to Peruvian 6- to 8-mo-olds daily for 7 mo was also not associated with caregiver-perceived appetite or growth, although the authors did find that the prevalence of diminished appetite before the intervention was a strong predictor of diminished appetite during the intervention (36). However, a study in 6- to 12-mo-olds in Ethiopia who received 10 mg Zn 6 for d/wk for 6 mo did show improved growth, especially among those who were stunted. They also showed morbidity and anorexia reductions associated with the intervention and postulated that the effects on growth were via these pathways (45). We offer a comparison of these studies utilizing a similar intervention because the variable results emphasize the need for a valid measure of appetite that can be adapted and used in multiple contexts. The absence of a significant change in appetite may be due to a true lack of improvement in appetite or to measurement methods that are not sensitive to real appetite changes and do not correlate with changes in growth. These 4 zinc-supplementation trials particularly highlight the need for a sensitive and validated assessment of appetite. Indeed, only 1 of these 4 studies used an appetite ascertainment method that had undergone a true validation assessment (36).

### Validation of appetite assessment methods and tools

Four distinct tools were reported by authors to have been validated in 6 studies (20, 23, 25, 27, 31, 42). An additional study conducted testing consistent with a validation but did not specify as such (36). Most author-reported validations compared actual energy intake during an observed feeding of a test meal with participants’ average daily energy intake based on caretaker record. For example, 3 of the 6 studies that used a related observed feeding at breakfast methodology compared it with total daily energy intake calculated from caretaker 24-h recall of foods consumed (31) or from weighed food records over 3 consecutive days (23, 25). The validation assessment by Oelofse (31) did not show a significant correlation between the average energy intake from the observed breakfast feeding and mean daily energy intake measured by caregiver 24-h dietary recall. However, another assessment of the tool estimated daily energy intake from researcher-measured food consumption identified some significant correlations, particularly when validated against energy intake of single meals assessed over multiple days. In a group of 4 studies published in one article, the correlation between energy intake from the test meal and energy from parent-reported breakfast intake ranged from 0.40 to 0.52, with variable significance (25). In a larger study, the energy intake of the test food was significantly correlated with 3-d observed breakfast energy intake both before (*r* = 0.49, *P* = 0.002) and after (*r* = 0.42, *P* = 0.008) zinc supplementation. Correlations between the test food energy intake and the 3-d, 24-h energy intake based on parental records were weaker and not significant compared with the breakfast-only intake before (*r* = 0.22, *P* = 0.18) and after (*r* = 0.26, *P* = 0.12) zinc supplementation (23).

In Iran, an item-retention process was used to calculate the Spearman correlation between a general appetite assessment (measured on a 1–21 scale) and a set of questions across domains that included the following: *1*) rate of eating, *2*) proportion of food consumed, *3*) range of places in which child will eat, and *4*) amount of food generally consumed. Each of these 4 questions was measured on a 1–5 scale, to create a 4–20 scale. The authors initially tested a fifth question in this set that measured the “variety of foods a child accepts;” however, this item was eliminated due to a lack of statistical correlation with the general appetite assessment question. Cronbach's α was calculated to measure the internal consistency for the final 4-domain retained items in the scale (α = 0.72) (42).

Among the assessments based on caretaker perceptions, only one study attempted to validate its questionnaire, a single-item questionnaire, against observed dietary intake (20). The authors conducted an intensive assessment of energy intake on the basis of 10 d/child of observed feeding over the course of an entire year. The authors observed children over 12 daytime hours and estimated nighttime intake. They also applied their single-caretaker perception of appetite question with this same frequency, and then validated the perception of appetite assessment against actual energy intake. Through documenting that reduced energy intake corresponded with reported anorexia, their results indicated that mothers can reliably identify poor energy intake, especially from nonbreast-milk sources. They were also able to correlate maternal perceptions of appetite with illness events, specifically febrile and diarrheal illnesses, although these episodes only explained 15% of anorexia episodes. Interestingly, the accuracy of maternal perceptions of appetite was not associated with socioeconomic factors such as quality of housing or maternal education (20). The assessment methodology of Khademian et al. (27) did not appear to be explicitly validated in the study context; however, the authors used a previously validated tool (the CEBQ). The authors described their “validation” of the questionnaire as a “supervised translation process into Persian” and did not describe any additional assessment of the tool in relation to another measure of appetite or dietary intake (27).

## Discussion

Within global development and health initiatives, there is a growing appreciation that the causes of undernutrition, especially stunting, are multifactorial and require both nutrition-specific as well as nutrition-sensitive interventions that address underlying determinants of undernutrition, including health services, the care environment, and food security (47). There is also an increasing interest in identifying and testing interventions to address the biological causes of poor growth, including infection, EED, and other causes of metabolic dysregulation, hormonal disruption, immune activation, and inflammation (48). The diminished appetite that results from a chronic proinflammatory state could serve as an efficient screening tool for biomedical causes of undernutrition, especially stunting.

The tools and measures that were identified in this review were quite variable and the studies show the wide range of purposes for measuring appetite among children in LMICs. Multiple-day parental recording of weights of specific foods offered minus weights of the exact foods remaining offer a way to calculate energy intake, but these methods are cumbersome and time and resource intensive. Direct observation of a test food appears to be the generally preferred method for measuring appetite. Indeed, the WHO “appetite test” is part of standard recommendations for determining whether an acutely wasted child can be treated on an outpatient basis (14). We did not include the WHO appetite test in our review because, to our knowledge, this test has not yet been validated specifically in a comparative study. However, the WHO “appetite test” is a measurement of the amount of an RUTF consumed based on the child's weight (49) and one study included in this review used a similar approach (21). Of methodologies that used direct observation of feeding of a test meal, the type of food consumed, the duration of observation of the feeding, and preconsumption fasting protocols varied considerably. Although not all studies that sought associations between observed test meals and energy intake on the basis of multiple-day dietary intake found significant results, there is reasonable validation data to support directly observed test meal feedings as a satisfactory method, albeit time and resource intensive, for assessing appetite.

Caregiver perceptions of their child's appetite might offer a more rapid and efficient method to assess child appetite. Indeed, although we identified several studies that attempted to interpret caregiver perceptions to assess a child’s appetite, the methods used were quite variable, and typically only relied on a single question [which sometimes only allowed dichotomous response options (i.e., yes or no, good or not good)]. Only 1 single-question tool was tested for validation against actual energy intake: Brown et al. (20) rigorously assessed and validated their caregiver appetite question. Unfortunately, when Stoltzfus et al. (41) attempted to use the same single question in Zanzibar, but with a 5-point instead of a 3-point scale, they found a very skewed distribution of responses that classified >80% of children in the top fifth category as having a “very good” appetite, thereby limiting their ability to accurately discriminate more subtle levels of suboptimal or poor appetite. They postulated that mothers were reluctant to report anything “bad” about their children and that they had a difficult time applying a Likert-type scale to the concept of appetite. Although their deworming intervention showed an association with improved appetite, anemia, and growth, they questioned the accuracy of the appetite findings due to concerns about the tool's face validity and social-desirability bias, and called for more-informative approaches to assess appetite. Notably, Brown et al. (20) (15%), Arsenault et al. (36) (19%), and Stoltzfus et al. (41) (16%) reported a similar prevalence of unhealthy appetite among children. These studies raise a question as to how many response options are needed to accurately capture healthy or good appetite compared with unhealthy or bad appetite.

On the basis of these findings, there are several possible approaches to inform improved rapid and efficient assessments of child appetite. First, although appealing because of its efficiency, a single question addressed at a single time point to one caretaker may not be sufficient. Appetite is a complex phenomenon, and any tool may need to leverage a broad span of domains. Multiple assessment domains provide an opportunity to understand the variety of factors associated with appetite, such as how current intake compares with usual intake, but also to understand more nuanced aspects of how appetite may be diminished. For example, “diminished appetite” in the reviewed studies refers to whether weaning food frequency is reduced (45), whether children prefer only certain foods or a reduced variety of foods (22, 42), and whether breastfeeding frequency, intensity, and duration were reduced (45). Second, formative research and pilot testing during tool development are critical steps in producing rigorous tools and were infrequently reported in the studies that we reviewed. Formative research is needed to establish face validity (i.e., that concepts are understood by respondents as measuring the topic that is intended to be measured), as well as content adequacy or validity (i.e., the extent to which a measure represents all aspects of a construct) (50). One common method for assessing the adequacy of questionnaire content is to ask respondents to categorize or sort items on the basis of their similarity to construct definitions (51). To date, the studies that directly set out to measure appetite have applied little, if any, formative research and psychometric methods to the development of their measures. The one exception to this is the CEBQ tool that was developed with the use of extensive psychometric methods, and adapted for use in Iran (27). However, although the CEBQ does measure appetite-related domains (e.g., enjoyment of food, food fussiness), this instrument is primarily designed to measure eating behaviors in the context of childhood obesity and eating disorders (43, 52). Relatedly, cultural and linguistic validation (not simply translation) is critical to ensure valid application in specific settings and adaptation across a variety of settings. Third, we endorse the application of procedures to validate against a more robust and accepted assessment of appetite, such as a multi-meal quantification of energy intake, or against an observed feeding of a test meal. Many factors likely need to be considered to identify the biological context of a child whose appetite is being assessed, including breastfeeding status, nutritional (e.g., anthropometric) status, the presence of acute or chronic illness, and current therapeutic or dietary intakes.

The diminished appetite that results from a proinflammatory state offers a potential opportunity to efficiently screen for biomedical causes of undernutrition, especially stunting (53). The identification of children most likely to respond to specific interventions that target underlying causes of chronic undernutrition is critical for maximizing potential benefit and avoiding side effects among children who would not receive benefit from such treatments. Furthermore, targeting treatment in an interventional trial may diminish the risk of type 2 error, whereby a null result may occur due to the inclusion of study participants who would not benefit from the test intervention, thereby risking the false dismissal of a potentially effective intervention (54, 55). Indeed, many of the studies included in this review used an assessment of appetite as an outcome measure in an interventional trial. However, the validity of appetite results are called into question if they are based on questions that are not validated against energy intake in the study context, are not well understood by their participant population, or where cultural factors or social desirability limit accurate measurement (41).

Appetite is already commonly assessed as an outcome in intervention studies, including as an intermediate outcome in the pathway to improving growth. We identified 4 studies that assessed zinc supplementation's effect on appetite (27, 36, 45, 47). However, these studies showed conflicting results for the effect of WHO zinc on appetite. These discrepant results may be due to differences in the intervention (even among similar interventions, dosing and duration varied), a lack of intervention effectiveness in the study population, or the use of an appetite measure that lacks the accuracy to identify more subtle ranges of poor appetite.

Strengths of our review include a comprehensive and systematic assessment of studies from a wide range of settings that used objective or subjective assessments of appetite among children across an illness and linear and ponderal growth–faltering spectrum. Our study is limited by several factors. First, the majority of validated measures that assessed appetite through direct dietary observation came from a related methodology and author group. Second, our article elimination methodology did not track the specific exclusion reason for each article from the initial search pool. On the basis of clear exclusion and inclusion criteria, we are confident that the retained articles reflect a comprehensive pool of studies that assess appetite in children in an LMIC context between 1995 and December 2016. Third, due to the lack of a consistent definition for child appetite, we retained studies that met our inclusion criteria regardless of how appetite was defined, resulting in appetite measures that assessed a broad array of appetite-related behaviors. These limitations underscore the need for more consistent definitions and robustly validated tools in a variety of contexts. Fourth, our review did not include biomarkers as a method of validating the tools against clear physiologic abnormalities. The regulation of appetite through endocrine signals, especially in the context of malnutrition where endocrine signaling becomes disrupted, is an important and evolving area. Serum biomarkers, such as ghrelin, glucagon-like peptide 1, oxyntomodulin, or leptin might prove to be useful assessments of appetite or standards for validation of appetite-assessment methods (56–59).

On the basis of this review, it appears that there are 2 broad potential approaches for accurately assessing child appetite in either home-based or clinical settings: directly observed test meals or multiple-day assessments of energy intake on the basis of food consumption. Age-appropriate, culturally acceptable, neutral, easily available, and commonly palatable foods should be used for test meals, so that they are eaten to appease appetite (rather than for novelty) and accepted by the majority of children (60). Although the protocols for these types of assessments have been adapted and replicated in a few contexts, there is much less consistency in how appetite is measured with the use of subjective, questionnaire-based approaches. Most of these approaches lack robust validation to dietary intake and a clear description of how they were adapted to their specific contexts. However, because direct feeding observations and test meals are time and resource intensive and not feasible in many clinical and community-based settings for diagnostic or screening purposes, questionnaire-based appetite assessments hold potential as a practical approach in both research and clinical contexts.

## Conclusions and Recommendations

Measures such as direct feeding observations are likely to provide the most accurate point-in-time assessment of children's appetite, but they are labor and time intensive. Thus, in addition to objective dietary intake measures, the clinical and research nutrition community would benefit from a validated questionnaire-type appetite assessment to efficiently screen appetite in children. Given the strong cultural influence on caregivers’ perception and interpretation of appetite, it is important that appetite-assessment tools be developed and validated in the cultural context that they will be used. Future appetite assessments should consider multiple purposes for child appetite measurement—especially the identification of reduced appetite among children with growth faltering or who are at risk of growth faltering but who are not yet classified as malnourished by anthropometric measures. These children may benefit from interventions other than, or in addition to, food-based approaches to address undernutrition, and from monitoring appetite response to interventions.

## Supplementary Material

Supplement Data

## Notes

Supported by a grant from the Bill and Melinda Gates Foundation (OP1136759).

Author disclosures: SBI, MH, GR, ML, KR, BN, TA, JW, and DMD, no conflicts of interest.

Supplemental Table 1 is available from the “Supplementary data” link in the online posting of the article and from the same link in the online table of contents at https://academic.oup.com/advances/.

Abbreviations used:

CEBQ: Child Eating Behavior Questionnaire
EED: environmental enteric dysfunction
LMIC: low- and middle-income country
RUTF: ready-to-use therapeutic food.
