# Supplementary material for: Systematic Review of Tools and Methods to Measure Appetite in Undernourished Children in the Context of Low- and Middle-Income Countries
Source: Adv Nutr. 2018 Nov 21;9(6):789–812. doi: 10.1093/advances/nmy042 (PMC6247147; doi:10.1093/advances/nmy042)
Supplement: Supplement Data [file nmy042_supplemental_data.pdf]

Online Supplemental Material

Online Supplemental Table 1.

Database search terms and expressions

CAB Direct (863 references)
(appetite OR hunger) AND ("child nutrition" OR children OR preschool OR infants) AND ("Developing Countries" OR "Threshold Countries" OR "Least Developed Countries") AND Yr:[1995 to 2016]

EMBASE
http://hsl.uw.edu/databases/embase

'appetite'/exp OR 'hunger'/exp) AND ('developing country'/exp OR 'africa'/exp OR 'south america'/exp OR 'asia'/exp OR 'central america'/exp)
Age Filter for Infant and Child

Global Index Medicus databases (WHO Regional Databases) (http://www.globalhealthlibrary.net/php/index.php)
Methods: Independent search strategies were developed for each of the 5 regional indexes that are part of the WHO Global Index Mediicus. For each database, the index was searched to identify words associated with “appetite”, “measurement”, and “child.” Relevant terms were included in the initial search. However, including words from each of these categories resulted in a limited number of articles. The search strategy was then broadened to only specify 2 of the 3 categories. The search terms for each index are listed below. The resulting abstracts were then reviewed by 2 independent reviewers for inclusion and exclusion. Results were compared and there were no discrepancies.

AIM (18 references)
Go to Advanced Form to enter this search:
malnutrition OR nutritional status OR nutrition assessment OR nutrition disorders OR hunger [Descriptor]
AND
child, preschool OR infant [Descriptor]

LILACS Search #1 (20 references)
"APPETITE" or "APPETITE disorders" OR "HUNGER" [Subject descriptor]
and

"CHILD" or "CHILD, preschool" or "INFANT" [Subject descriptor]
and

"MEASUREs" or "evaluation of research programs and INSTRUMENTs" or "evaluation of research programs and TOOLs" or "health situation ASSESSment" or "nutrition ASSESSment" or "QUESTIONNAIREs" or "SURVEY methods" or "SURVEYs" or "nutrition SURVEYs" or “SCALEs” [Subject descriptor]

LILACS Search #2 (2 references)
nutrition assessment [Subject descriptor]
and
("CHILD" or "CHILD, preschool" or "INFANT") [Subject descriptor]
and
appetite OR hunger OR hungry [Words]

IMSEAR (19 references)
Search by Title Contains: measure OR assess OR evaluate OR questionnaire OR survey OR tool OR instrument OR scale OR index
Filter: subject contains: appetite OR hunger OR “desire to eat”

Online Supplemental Material

WPRIM (62 references)
All: appetite OR hunger [Logic: AND]
All: measure OR measures OR measurement OR assess OR assessment OR evaluate OR evaluation OR questionnaire OR questionnaires OR survey OR surveys OR tool OR tools OR instrument OR instruments OR scale OR scales OR index OR indexes OR indices [Logic: AND]
All: child OR children OR preschool OR infant OR infants OR baby OR babies OR toddler OR toddlers OR pediatric OR paediatric

IMEMR (129 references)
appetite OR hunger [key words]
AND
measure OR measures OR measurement OR assess OR assessment OR evaluate OR evaluation OR questionnaire OR questionnaires OR survey OR surveys OR tool OR tools OR instrument OR instruments OR scale OR scales OR index OR indexes OR indices [key words]

PubMed Search #1 (449 references)
("Nutrition Surveys"[Mesh] OR "Nutritional Status"[Mesh] OR "Malnutrition"[Mesh] OR "Growth Disorders"[Mesh] OR "Nutritional Physiological Phenomena"[Mesh:NoExp] OR "Child Nutritional Physiological Phenomena"[Mesh] OR "Appetite Regulation"[Mesh] OR "Nutritional Requirements"[Mesh] OR "Thinness"[Mesh] OR undernourish*[tiab] OR malnutrition[tiab] OR malnourish*[tiab] OR underweight[tiab] OR stunt*[tiab] OR “Failure to Thrive”[MeSH] OR wasting[tiab] OR “weight for height”[tiab] OR “height for age”[tiab] OR “weight for age”[tiab]) AND (infant[MeSH:noexp] OR child, preschool[MeSH]) AND ((“Hunger”[mesh] OR hunger[tiab] OR Appetite[tiab] OR (desire[tiab] AND eat[tiab]) OR (desire[tiab] AND food[tiab]) OR anorexia[tiab])) AND (Test OR tests OR testing OR tested OR Tool OR Tools OR Measure* OR Instrument* OR Survey* OR Rating* OR Assess* OR Questionnaire* OR Scale OR scales OR Index OR indices OR "Epidemiologic Studies"[Mesh] OR "Epidemiologic Study Characteristics as Topic"[Mesh] OR "Clinical Trial" [Publication Type])

PubMed Search #2 (for the recent, unindexed references) (38 references)
((“nutritional status” OR undernourish*[tiab] OR malnutrition[tiab] OR malnourish*[tiab] OR underweight[tiab] OR stunt*[tiab] OR “Failure to Thrive”[tiab] OR wasting[tiab] OR “weight for height”[tiab] OR “height for age”[tiab] OR “weight for age”[tiab]) AND (child[tiab] OR children[tiab] OR newborn[tiab] OR childhood[tiab] OR baby[tiab] OR babies[tiab] OR toddler*[tiab] OR infant[tiab] OR infants[tiab] OR infantile[tiab] OR pediatric*[tiab] OR paediatric*[tiab]) AND (hunger[tiab] OR Appetite[tiab] OR (desire[tiab] AND eat[tiab]) OR (desire[tiab] AND food[tiab]) OR anorexia[tiab]) AND (Test OR tests OR testing OR tested OR Tool OR Tools OR Measure* OR Instrument* OR Survey* OR Rating* OR Assess* OR Questionnaire* OR Scale OR scales OR Index OR indices OR “clinical study” OR “clinical trial” OR “clinical studies” OR “clinical trials”) AND ("2015”[PDat] : "2016"[PDat])) NOT medline[sb]
